# The Roles of Regional Organisations in Strengthening Health Research Systems in Africa: Activities, Gaps, and Future Perspectives

**DOI:** 10.34172/ijhpm.2022.6426

**Published:** 2022-03-08

**Authors:** Catherine M. Jones, Joëlle Sobngwi-Tambekou, Rhona M. Mijumbi, Aaron Hedquist, Clare Wenham, Justin Parkhurst

**Affiliations:** ^1^Department of Health Policy, London School of Economics and Political Science, London, UK.; ^2^Recherche-Santé & Développement (RSD Institute), Yaoundé, Cameroun.; ^3^The Centre for Rapid Evidence Synthesis, College of Health Sciences, Makerere University, Kampala, Uganda.; ^4^LSE Health, London School of Economics and Political Science, London, UK.

**Keywords:** Regional Organisations, Regional Cooperation, Health Research Systems, Health Research, Health Sciences Research, Africa

## Abstract

**Background:** Regional cooperation on health in Africa is not new. The institutional landscape of regional cooperation for health and health research, however, has seen important changes. Recent health emergencies have focussed regional bodies’ attention on supporting aspects of national health preparedness and response. The state of national health research systems is a key element of capacity to plan and respond to health needs – raising questions about the roles African regional bodies can or should play in strengthening health research systems.

**Methods:** We mapped regional organisations involved in health research across Africa and conducted 18 interviews with informants from 15 regional organisations. We investigated the roles, challenges, and opportunities of these bodies in strengthening health research. We deductively coded interview data using themes from established pillars of health research systems – governance, creating resources, research production and use, and financing. We analysed organisations’ relevant activities in these areas, how they do this work, and where they perceive impact.

**Results:** Regional organisations with technical foci on health or higher education (versus economic or political remits) were involved in all four areas. Most organisations reported activities in governance and research use. Involvement in governance centred mainly around agenda-setting and policy harmonisation. For organisations involved in creating resources, activities focused on strengthening human resources, but few reported developing research institutions, networks, or infrastructure. Organisations reported more involvement in disseminating than producing research. Generally, few have directly contributed to financing health research. Informants reported gaps in research coordination, infrastructure, and advocacy at regional level. Finally, we found regional bodies’ mandates, authority, and collaborations influence their activities in supporting national health research systems.

**Conclusion:** Continued strengthening of health research on the African continent requires strategic thinking about the roles, comparative advantages, and capability of regional organisations to facilitate capacity and growth of health research systems.

## Background

**Key Messages**

** Implications for policy makers**

- Given the multi-sectoral composition of national health research systems, national policy-makers from the health, higher education, and science policy sectors may be individually unaware of various regional organisations’ involvement and activities where intersectoral coordination is lacking. Decision-makers across sectors may benefit from collectively identifying which regional organisations are supporting these systems and assessing in what ways.
- National governments may need to strengthen the mandates of regional bodies and grant them additional authority to realise the potential gains these bodies can achieve in terms of governance and coordination of health research.
- Several opportunities exist for regional bodies to contribute to improving health research in Africa. There are particularly notable gaps in financing of health research and advocacy, which regional organisations may be strategically placed to address in the future.

** Implications for the public**

Health research systems are important to help governments plan and improve health services for the public and to respond to new crises and outbreaks. Research in the health sector also can be a potential driver of economic activity, innovation, and growth. Yet many African countries still struggle with capacity in this important area. Regional organisations that are made up of and serve multiple member states across the African continent can play important roles in strengthening and coordinating health research at national levels. This research helps understand the ways these regional bodies are working to do so, and important gaps that could be addressed in the future. It also proposes future directions for research to contribute to empirical exploration of narratives on regional cooperation for health research.

Health is increasingly gaining attention from regional organisations, alongside more traditional aspects of regional cooperation like trade or security,^1^ with regional organisations in the global south seen to be important policy venues within multi-level governance of health.^2^ However, the way that health policy is framed and understood as an issue for regional cooperation varies across organisations and has been shown to be influenced by context-specific social, economic, and political views on health policy by member states.^3-5^

In Africa, regionalism has been influenced by international trade and economic interests, colonial histories, alignment of social and economic policies, and disease threats to public health – although health is not generally the core interest for organisations in regional governance.^6,7^ Recent health crises, however, have increased attention to the role of regional bodies in health sector planning and response. The 2014 Ebola outbreak in West Africa, for instance, highlighted the importance of regional organisations in health emergencies as well as their involvement in strengthening public health and health systems in the global south.^8,9^ Currently the coronavirus disease 2019 (COVID-19) pandemic similarly highlights the importance of regional actions to improve health preparedness and response in Africa.^9,10^ It has also underscored the critical contributions of regional cooperation to legitimize the experience of states and health systems with outbreaks across the continent, to network national decision-makers, to share information, to improve infrastructure such as laboratories, and to use research capacity of African scientists and institutions for local knowledge to inform and plan health responses.^11-13^

National health research systems serve as a key component of a state’s ability to respond to both acute and long-term health needs.^14^ They, however, have also been seen to provide key economic development opportunities through specialised areas of expertise, high-tech employment, and innovation. Thus, we need better understanding and answers to an important question: what is the role of regional bodies in strengthening health sciences research (HSciR) and health research systems within and between countries in Africa? HSciR includes fundamental, clinical, applied, and implementation research on human health and well-being, as well as the determinants, prevention, detection, treatment, and management of disease.^15,16^

 The landscape of regional cooperation for health and HSciR in Africa has undergone key institutional changes at the continental level in recent years. For example, the launch of Africa Centres for Disease Control and Prevention (Africa CDC) in 2017 (the public health agency of the African Union [AU]), and the transformation of the New Partnership for Africa’s Development Planning and Coordinating Agency (NEPAD Agency) into the African Union Development Agency (AUDA-NEPAD) in 2019 have been two key changes. There have also been several programmes initiated at the regional level to improve the funding of research and development, including HSciR, towards meeting goals of the Science, Technology, and Innovation Strategy for Africa 2024 of the AU. Despite signposts of strategic activity in African regionalism for health, the roles of regional organisations in HSciR have not been extensively documented or analysed.

One important reason to explore their role is because regional cooperation may support opportunities for equitable improvement of HSciR performance between countries in Africa.^17^ Research on national health research systems found that countries which showed higher performance on metrics such as publications and trials, as well as greater human resources and institutional capacity, have generally benefited from substantial, long-term international partnerships and collaborations.^18,19^ Yet relying on international funding and partnerships to develop HSciR at a national level risks generating inequalities between countries. Regional cooperation has been seen to be a useful approach to reduce inequalities in research capacity of national health research systems. For example, the development of a regional laboratory can bring efficiency gains to countries with little or no national research infrastructure.^20^

Regional organisations could therefore, in theory, support a coordinated approach to strengthening HSciR that reduces disparities in HSciR between African states whether through regional financing schemes, building regional centres of excellence,^21^ or promoting knowledge and technology transfer between African countries. Currently, little is known about what is already being done by regional organisations in Africa in this area. Drawing extensively from a research report,^22^ this paper therefore aims to understand what regional organisations are doing to strengthen HSciR and health research systems in Africa.

###  Conceptual Approach

**Figure 1:**
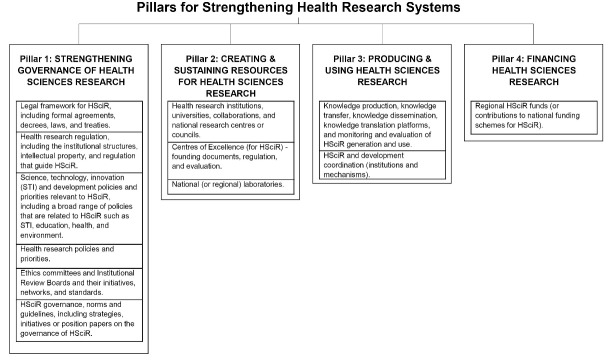
Pillars for Strengthening Health Research Systems. Adapted from Pang et al^23^ and Kirigia et al.^28^ Abbreviation: HSciR, health sciences research.

While the literature identifies some examples of regional bodies influencing HSciR in Africa, we explore this more systematically by considering the key elements typically held to be central to state HSciR capacity. To do this we utilise the four key pillars of health research systems: governance, creating and sustaining resouces, producing and using research, and financing.^23^ These pillars and their sub-elements can be seen in Figure 1.

The pillars have been widely adopted to evaluate the functions of health research systems (see ^24-26^). Indeed, the World Health Organization Regional Office for Africa (WHO AFRO) has formulated the targets in its regional strategy for health research in Africa around these essential pillars and has been regularly assessing and monitoring the development and progress on each of them.^27-32^ Consequently, the pillars provide a comparable, established, and justifiable framework to consider how regional cooperation may contribute to strengthening HSciR in countries and national health research systems. Specifically, we explore which activities reportedly undertaken by regional bodies could support each pillar, while also reflecting on what regional stakeholders think regional bodies can or should be doing in relation to each pillar – to further identify gaps in areas of activity that may be particularly useful for regional bodies to address.

## Methods

We mapped regional organisations across Africa involved in HSciR and conducted 18 interviews with key informants from 15 of them. We defined regional organisations as those with membership of at least three countries within in any of the five regions of the continent defined by the AU (https://au.int/en/member_states/countryprofiles2), or with membership spanning more than one region. We used this definition to emphasise the importance of regional organisations comprised of member states as distinguished from regional networks, associations or consortia. The former are likely to interact directly with representatives of government institutions as key policy stakeholders for national health research systems, whereas the latter are more likely to have relationships with individual researchers, research institutions or labs, practitioners, or non-governmental organisations.

The mapping exercise aimed to identify African regional bodies active in at least one of the key pillars illustrated in Figure 1. We began with a list of key regional stakeholders identified from previous research on national health research systems^18^ and all regional economic communities of the AU (https://au.int/en/recs). We further canvassed members of expert networks active on the continent to identify additional organisations. AH conducted a manual search of all organisations’ websites and their governing, strategic, and policy documents to identify HSciR-related activity or stated impact in any of the four pillars. Organisations’ documents were also used to identify additional regional bodies for the list. Documents from newly identified organisations were then searched, and the process continued until no new organisations were identified.

We included regional organisations if they met our broad definition of an African regional organisation, had activity related to at least one of the key pillars, and if they were not extensively governed by members outside the African continent. Following analysis by AH and CMJ, and in-depth discussion with JST and RM, the final stakeholder map was validated by all authors. It included 22 main regional organisations (with a total of 45 including their relevant sub-organisations) (see Supplementary file 1for list). The African Academy of Sciences (AAS) was the only organisation we included which did not meet the eligibility criterion according to our definition of regional organisations. Our decision to include it as the only non-member state based regional organisation in the study was justified by its relevance at the time of study as a regional science funding platform mandated by NEPAD for this purpose in 2015. We judged this decision acceptable given consensus among partners and expert networks about the unique position of AAS with its mandate expanded in collaboration with an AU agency to accelerate and fund excellence in African science, including HSciR^[1]^. For each regional organisation, AH collected the following data from their websites: relevant sub-organisations, headquarters location, official mandate for regional cooperation, geographic sub-region of the organisation’s mandate (according to the AU’s five regions), member states, description of the organisation’s role in supporting HSciR, and links to HSciR specific activity (eg, a programme, project, policy). Organisations were then categorised by their primary mandates in economic, political, or technical cooperation, as summarised in Table 1.

**Table 1:** Types of Regional Organisations

**Economic**	Organisations mandated to improve the economic situation of African States, such as trade organisations and economic cooperation groups.
**Political**	Organisations mandated to regulate and negotiate political relationships between nations within Africa, such as multilateral organisations and other normative institutions.
**Technical (Development)**	Organisations mandated to provide technical expertise, support, and/or coordination of development activities or policy.
**Technical (Education)**	Organisations mandated to provide technical expertise, support, and/or coordination of higher education activities or policy.
**Technical (Health)**	Organisations mandated to provide technical expertise, support, and/or coordination of health activities or policy.
**Technical (Science)**	Organisations mandated to provide technical expertise, support, and/or coordination of science activities or policy.

AH, in collaboration with CMJ, analysed data from the mapping exercise by looking for either evidence of involvement or declared goals or intentions of involvement in HSciR based on information and documents from websites. Decisions about the classification of organisations according to whether they had evidenced (explicit), declared (implicit), or expected (but unseen) interest in HSciR were made collectively by AH, CMJ, JST, and RM. CMJ, JST, RM, CW, and JP prioritised organisations for interviews if they had evidenced or declared interest based on results from our mapping (see Supplementary file 2 for list of priority organisations). But we also considered expert opinion from members of the research team (JST and RM) and other stakeholders with knowledge of regional organisations to select priority organisations, given that documents of evidenced or declared interest are not always available online (eg, websites not up to date, several relevant documents may only exist in hard copy). Our selection of priority organisations also ensured inclusion of Francophone bodies, geographic representation, and different organisational types. RM, JST, and CMJ invited the 19 regional organisations (2 of the 20 on the list in Supplementary file 2 are sub-organisations from the East African Community) from the mapping for interviews to investigate in greater depth their activities, impact, and potential for HSciR development within countries. In total, we invited 39 individual informants. Throughout the recruitment process, we identified the most relevant informants for interviews in dialogue with key contacts in these organisations. Only one informant declined to participate stating that their organisation did not do anything in the area of HSciR; however, one informant was unable to find any availability in their agenda for an interview despite expressed interest and multiple attempts to accommodate their schedule and several (n = 7) did not reply to any email or telephone contact, including instances when they were recommended by internal colleagues.

The data analysed for this paper come from 18 interviews (13 in English, 5 in French) conducted remotely mainly by JST, RM, and CMJ, with informants from 15 regional organisations (see Table 2) between January and April 2021. Half of the informants were in senior technical or operational positions, and half were in executive and strategic posts. The structured interviews asked about the roles, challenges, and opportunities of these bodies in strengthening HSciR across the continent, within and between countries (see Supplementary file 3for interview guide).

**Table 2:** Organisations Interviewed

**Description of Regional Organisations**					**Health Research System Pillars**			
**Expertise**	**Organisa**t**ion**	**Year Founded**	**Organisation Type**	**Internal Governance**	**Governance**	**Resources/Infrastructure**	**Production/Use**	**Financing**
Economic	ECCAS	1983	Regional Economic Community	Intergovernmental/Member States				
Technical (higher education)	CAMES	1972	Continental Organisation	Intergovernmental/Member States				
	IUCEA	1980	Regional Economic Community	Hybrid membership (Member States + universities)				
Technical (development)	AfDB	1964	Multinational Financial Organisation	Governing Board				
	AUDA-NEPAD (*NEPAD secretariat became NEPAD Agency in 2010; AUDA-NEPAD established 2019)	2001*	AU Agency/Continental Organisation	Intergovernmental/Member States				
	IGAD	1986	Regional Development Community	Intergovernmental/Member States				
	SRO-EA/UNECA	1958	UN Agency	Intergovernmental Think Tank/Member States				
Technical (health)	Africa CDC	2017	AU Agency/Continental Organisation	Governing Board				
	ECSAHC	1974	Inter-regional Health Community	Intergovernmental/Member States				
	OCEAC	1963	Regional Health Organisation	Intergovernmental/Member States				
	WAHO	1987	Regional Health Organisation	Intergovernmental/Member States				
	WHO AFRO	1965	UN Specialised Agency	Regional Committee/Member States				
	WHO EMRO	1949	UN Specialised Agency	Regional Committee/Member States				
Technical (science)	AAS (*Accelerating Excellence in Science in Africa established 2015)	1985*	Continental Organisation	Non-State Actor/NGO				
	ARIPO	1976	Continental Organisation	Intergovernmental/Member States				

Abbreviations: ECCAS, Economic Community of Central African States; CAMES, Conseil Africain et Malgache pour l’Enseignement Supérieur; IUCEA, Inter-University Council for East Africa - East African Community; AfDB, African Development Bank; AUDA, African Union Development Agency; NEPAD, New Partnership for Africa’s Development Planning and Coordinating; IGAD, Intergovernmental Authority on Development; SRO-EA/UNECA, Subregional Office for Eastern Africa, United Nations Economics Commission for Africa; Africa CDC; Africa Centres for Disease Control and Prevention; ECSAHC, East, Central, and Southern Africa Health Community; OCEAC, Organisation de Coordination pour la lutte contre les Endémies en Afrique Centrale; WAHO, West African Health Organisation; WHO, World Health Organization; AFRO, Africa Regional Office; EMRO, Eastern Mediterranean Regional Office; AAS, African Academy of Sciences; ARIPO, African Regional Intellectual Property Organization; AU, African Union; UN, United Nations; NGO, Non-Governmental Organisation.

JST, RM, and CMJ deductively coded the interview data according to the key pillars for HSciR that regional organisations are involved in; how they are carrying out this work; and in which pillars they perceive they are having impact (see Supplementary file 4for code book). CMJ then carried out a comparative analysis of the activities across all the organisations, seeking to understand the advantages different regional organisations have in working in particular areas; the gaps in activities; and common themes of barriers and facilitators for regional organisations working to support HSciR in Africa. Questions arising from the comparative analysis were discussed in-depth together with JST, RM, CW, and JP to validate the results collectively as a research team.

###  Strengths and Limitations

There are several strengths of this study. The use of semi-structured interviews allowed us to query the perspectives and access the experiential knowledge of key informants working in regional organisations in Africa. These data are qualitatively different from information that would be gathered from documents or websites because the interview allows for follow-up, probing, and exploration of specialist and insider knowledge about the regional organisation, which is well documented knowledge about interviews with decision-makers.^33^ It is through methods of collecting data from interviews with senior executives and managers in regional organisations that we could best answer our research question about their role (current, future, and gaps) in HSciR strengthening and gain insight into their perceptions of positionality and impact within a regional or national landscape to support health research systems. As such, our methods explicitly intended to collect data beyond publicly available communications such as websites and organisational documents to question not only whether regional organisations were involved in HSciR strengthening (or not), but how they were involved. The website content and policy documents to support our selection of priority organisations was generally limited to the continental organisations and rather scarce or absent for many regional communities, despite their involvement as per our interview data.

 The findings should be considered within potential limitations related to the representativeness of the organisations interviewed. Despite a rigorous and multifaceted informant recruitment process as detailed above, several organisations and regional economic communities are missing from our interview data. We do not think this is a major limitation, as a comprehensive picture of every regional organisation’s role is not essential to meet our research objective. Furthermore, we are not making claims of generalisability of our findings to all regional organisations in Africa. Although we did not interview all prioritised organisations, the representation of a mix of regional organisation types and expertise make our findings on comparative advantages and common themes relevant as a purposive sample across this range. However, different strengths or gaps may have been identified by speaking with additional regional economic communities, and other technical organisations like the East African Health Research Commission, which has a specific mandate in health research.

## Results

Most organisations interviewed stated being active in two or more pillars, the most frequent of which were governance and producing and using research. We found differences in involvement according to expertise by type of organisation. The organisations which reported being active across all four pillars were generally those with health or higher education expertise. The financing pillar had the least amount of reported activity from the organisations in our study. Table 2 provides an overview of the 15 organisations. It presents details of each organisation, and which pillars we identified where at least one activity was undertaken. This is not weighted to represent volume of activity; nevertheless, it paints a broad picture of where different organisations’ efforts fit into a framework of improving core functions of health research systems.

**Figure 2:**
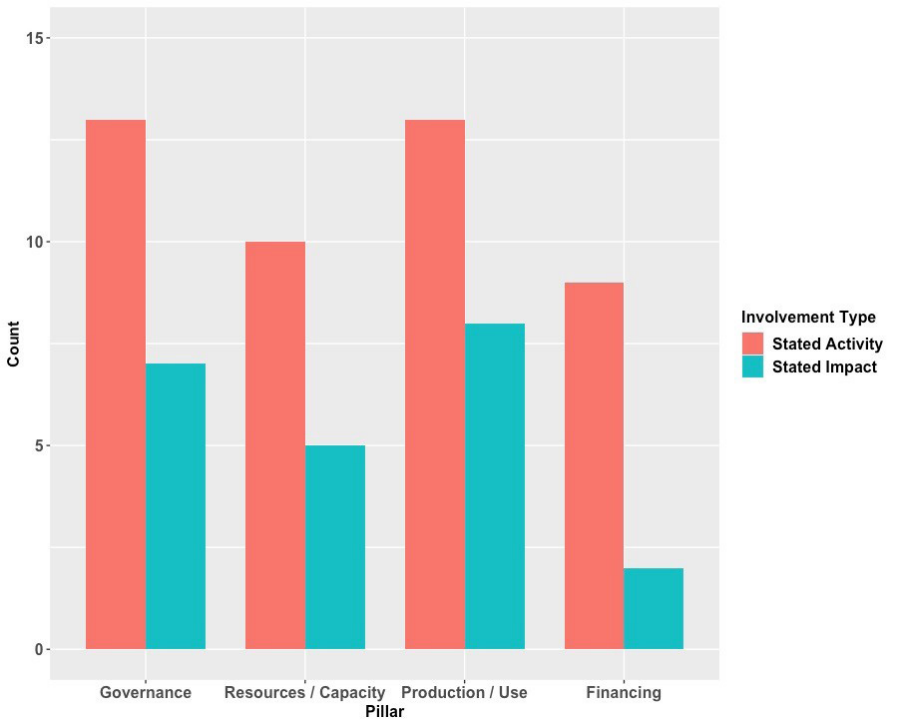
Activities and Impacts Health Sciences Research by Pillar for all Regional Organisations Interviewed.

Looking at each pillar on an aggregate level, we also found important differences within our sample when we compared regional organisations’ reported activities against those where they felt they achieved impact. Figure 2provides this comparison.

###  Governance of Health Sciences Research

 The governance pillar refers to the policy and legal frameworks and institutional structures to steer and manage HSciR, including ethical governance. Most of the organisations we interviewed reported being involved in supporting the governance of HSciR in Africa in some way. However, Africa-CDC and WHO regional offices were the regional organisations which appeared to have the most authority and leadership in this area across the continent. We found the activities carried out by regional organisations involved in HSciR governance to include agenda-setting and strategy development, the provision of guidance for national governance of health research (including research ethics), the harmonisation of policies within regional communities, and the coordination of national health research at the regional level. Overall, these activities seem to focus on regional integration of policies, regulations, priorities, standards, and norms linked to HSciR. According to informants’ own perceived impact of their organisations, their activities in this pillar appeared to have been successful within sub-regions, especially when they are led by bodies with technical expertise, in health or higher education.

 The main mechanism by which regional organisations are involved in governance of HSciR at the national level appears to be through their efforts to support harmonisation of national policies across four policy areas: pharmaceutical policy, public health policy, higher education policy, and intellectual property (IP) policy. According to our interviews, policies on medicines and therapeutics are one of the key regulatory policy domains which regional organisations focus their harmonisation efforts on to improve inspection, approval, and use of high-quality and affordable medicines. For example, Organisation de Coordination pour la lutte contre les Endémies en Afrique Centrale (OCEAC) has developed a common pharmaceutical policy across its 6 member states, and West African Health Organisation (WAHO) uses a single medicine registration process for all 15 of its member states. Many organisations have worked together towards the establishment of the African Medicines Agency by the AU (the second AU health agency after Africa CDC), which was formally in November 2021 as the body responsible for regulatory systems for medicines and medical products in Africa. Many informants expressed high expectations for this continental agency, whose antecedent was the African Medicines Regulatory Harmonization initiative by NEPAD implemented by regional economic communities with technical support from WHO. Some organisations (eg, WAHO, OCEAC, Intergovernmental Authority on Development [IGAD], Africa CDC) also stated activities to harmonise public health policies (eg, guidelines for tuberculosis, malaria, family planning) and medical practices across their member states. Similarly, regional bodies specialising in higher education (eg, Conseil Africain et Malgache pour l’Enseignement Supérieur [CAMES] and Inter-University Council for East Africa - East African Community [IUCEA]) reported focusing their involvement in HSciR governance on harmonising policies and standards for academic professional development in universities and evaluation criteria for education quality. For instance, in 2017 East African heads of state declared the sub-region a common higher education community to support integration of higher education policies and standards in various fields (including medicine and health sciences). In the domain of IP, African Regional Intellectual Property Organization (ARIPO) promotes harmonisation of IP rights and laws within member states across multiple sub-regions.

We also found a group of intergovernmental organisations that are more specifically trying to set the agenda for strengthening HSciR at the continental level. Organisations such as AUDA-NEPAD, WHO Africa Regional Office (AFRO), and Africa CDC have developed strategies intending to establish a shared policy framework for countries to adapt and align their own HSciR policies and programmes for health research systems.^27,34^ WHO AFRO also has an internal mechanism (the African Advisory Committee on Health Research and Development) to advise the Regional Director General on monitoring implementation of regional policy on health research and on supporting countries to improve their national health research systems.

 The final activity carried out by regional organisations under the heading of HSciR governance is the coordination of national health research activities. Africa CDC is a unique technical organisation among those interviewed due to its mandate from the AU as a designated authority to coordinate the health research agenda and integrate research and analysis practices across the continent. Africa CDC’s approach to coordination differs from others included in our study because the headquarters works through its 5 regional collaborating centres that have relationships with their corresponding regional economic communities and with member states through national public health institutes (NPHIs). The strategic vision of Africa CDC is to have a NPHI in every African country to strengthen public health capacity (including research) links to its networked multi-level approach to coordination. This contrasts to the coordination apparatus of WHO regional offices, the other main technical organisation with a health mandate on the continent, with more political features of coordination with direct relationships to governments through Ministries of Health. One advantage of WHO, however, is the organisation’s presence in-country which, in theory, supports its coordination activities. However, informants underscored that WHO’s comparative advantage as a normative organisation is around technical assistance, guidelines, and evidence support with its coordination efforts between states and other regional bodies not being its main strength.

####  Key Gaps - Governance

 Multiple informants expressed coordination challenges due to limitations in member states’ willingness to collectively participate in policy harmonisation, even if it falls within the mandate of the regional organisation. From our interviews, informants perceived the greatest gap when it comes to continent-wide or inter-regional coordination. Few organisations seem to be coordinating across remits of multiple stakeholders at the continental level. The AUDA-NEPAD mainly coordinates with the regional economic communities, but not every bloc has an organisation with health expertise. Africa CDC coordinates with NPHIs via its regional centres, and WHO coordinates with Ministries of Health. While these AU and UN agencies may coordinate specific programmes (ie, African Vaccine Forum), there is no systematic general coordination happening, nor is any agency formally mandated with responsibility for that across regional communities or continental agencies. This siloed coordination poses a problem when regional bodies may not be liaising with the main institution mandated for governing the national health research system in a specific country.

###  Creating and Sustaining Resources for HSciR

 The resources pillar includes human resources (a critical mass of highly qualified researchers and research personnel with HSciR skills and competencies), institutional resources (universities and research institutions), and research infrastructure (labs, equipment). We found that organisations with mandates in health, higher education, or science which had activities related to HSciR governance were also typically involved in strengthening resources for HSciR. However, this was less seen for bodies with economic or development mandates.

We found more regional organisations were involved in promoting human resources and individual skills for HSciR, and fewer to be involved in strengthening research institutions and infrastructure. Table 3 provides a summary of the range of activities by regional organisations to strengthen resources and infrastructure for HSciR. Africa CDC, WHO regional offices, WAHO and CAMES were identified to be the regional organisations in our study carrying out the most comprehensive activities to strengthen resources. Africa CDC and OCEAC also reported supporting south-south collaboration and knowledge transfer by twinning universities and labs for training exchanges for researchers (eg, epidemiology, lab techniques). We found that organisations like WAHO and CAMES have supported networking between research institutions, but this was rare among the regional organisations in our sample.

**Table 3:** Summary of Regional Organisations’ Activities to Strengthen Resources and Infrastructure for Health Sciences Research

	**Skills Building (Curricula, Short Courses, Workshops)**	**Research Networks, Mobility, or Exchanges**	**Capacity Funding**	**Institutional Policies, Structures, or Mechanisms**	**Infrastructure (Laboratories, Equipment)**
AAS			Alliance for Accelerating Excellence in Science in Africa (multiple programs to support research capacity, infrastructure, and leadership)		
Africa CDC	Training in: field epidemiology, laboratory analysis, research ethics, scientific writing for publication, MSc in Epidemiology, MSc in Biostatistics			National Institutes of Public Health	Investment in national lab infrastructure, equipment, and supplies
ARIPO	• Training police officers to investigate IP crimes • Curriculum on building respect for IP rights and rules • Masters in IP			Providing universities and research institutions with: • models for patent applications • templates for institutional IP policy development and guidance	
CAMES				Guidelines and regional standards for: • promotion of researchers and faculty • accreditation of programmes • evaluation protocols for doctoral programmes • indicators for research training and pedagogy	
IGAD	Training for government institutions, government service providers, policy-makers, decision-makers				
IUCEA				Working with universities to improve graduate/post-graduate supervision	
OCEAC		Hosting student exchanges for laboratory training with university partners			Setting up research labs
WAHO	Training in: research ethics, scientific writing	Hosting secretariat for thematic research networks (maternal health, infectious diseases, child health, clinical trials)	• Capacity building fund • Commodities fund		Laboratory collaborations (eg, West African Biobank)
WHO AFRO	• Sharing standard research protocols • Workshops on: writing policy briefs, research methods, research ethics, scientific writing			Supporting Ministries of Health to include health research in mandate	
WHO EMRO	Workshops on: writing policy briefs, research methods, research ethics, scientific writing			Supporting Ministries of Health to include health research in mandate	

Abbreviations: CAMES, Conseil Africain et Malgache pour l’Enseignement Supérieur; IUCEA, Inter-University Council for East Africa - East African Community; IGAD, Intergovernmental Authority on Development; Africa CDC; Africa Centres for Disease Control; OCEAC, Organisation de Coordination pour la lutte contre les Endémies en Afrique Centrale; WAHO, West African Health Organisation; WHO, World Health Organization; AFRO, Africa Regional Office; EMRO, Eastern Mediterranean Regional Office; AAS, African Academy of Sciences; ARIPO, African Regional Intellectual Property Organization; IP, intellectual property.

 WHO AFRO and Africa CDC stand out in this pillar for their work across the continent with public institutions that make and implement decisions about HSciR. Their efforts seem complementary, with WHO working with governments through Ministries of Health and Africa CDC with the public health workforce for HSciR through research institutions and NPHIs. Working to improve national health research systems through its regional strategy for health research, WHO AFRO advocates that Ministries of Health incorporate health research as a health sector responsibility and use research to improve policies, programmes, and interventions. For example, throughout the COVID-19 pandemic, WHO AFRO has shared standard research protocols with member states to support rapid and rigorous knowledge generation across the continent. Furthermore, both of the WHO regional offices covering countries in Africa (AFRO and Eastern Mediterranean Regional Office [EMRO]) have carried out training to improve health research governance within countries, which they reported has led to the establishment of ethics committees in several countries.

 When it comes to funding programs for the development of human resources for research across the continent, AAS is a unique organisation among those in our sample. Designated by the AU as an advisory and implementation body for its Agenda 2063 (the AU framework for sustainable development) and the Science, Technology and Innovation Strategy for Africa (STISA-2024), AAS carries out several programmes with competitive grants to support training and development of individual researchers and networks through its Alliance for Accelerating Excellence in Science in Africa platform. At the sub-regional level, WAHO also reported facilitating resource development through specific funding programmes.

####  Key Gaps – Resources 

 Research infrastructure is critical within countries to have the physical environment, equipment, and material resources available for researchers. The majority of regional organisations did not report being involved in or investing in research infrastructure development in member states. While regional organisations for the most part do not fund health research infrastructure improvements, regional centres of excellence have been cited as opportunities for HSciR infrastructure development at the regional level. Regional organisations could also encourage and routinise HSciR infrastructure development as part of any investment in human resources development. Despite the human resource development interventions reported, the distribution of skilled researchers remains uneven across the continent. Multi-country teams have been set up by WAHO, AAS, and CAMES but more can be done by regional organisations to facilitate networking. As one informant emphasised, this is a particularly important role for regional health organisations to convene research networks that foster equity in research collaborations by including researchers from countries without research-active universities and/or insufficient faculty for research education and training in their respective regions.

###  Producing and Using Health Sciences Research

 The third pillar for strengthening HSciR refers to the production (research projects/programmes, publications) and use (dissemination, communication, translation) of knowledge. Like the governance pillar, most of the regional organisations we spoke to stated involvement in this pillar. But few organisations were found to be involved in knowledge production itself. Some technical organisations in health are conducting HSciR research in-house (eg, OCEAC, WAHO), but most are doing research with partners and consultants. Most of the activity reported in this pillar related to knowledge dissemination and translation. We found this to be a potential strong comparative advantage for regional organisations, which have the convening power and, in some instances, the official mandate, to bring together researchers (knowledge producers) and policy-makers (knowledge users) to discuss research uptake.

 Regional organisations are playing a role in knowledge dissemination and use in multiple ways. WHO EMRO and WHO AFRO have conducted training for evidence use in health policy and practice in countries that request it, including for drafting policy briefs for decision-makers. They also advocate to member states to set up evidence into policy networks as part of their normative role to work with Ministries of Health to strengthen national health research systems. The same regional organisations convene policy forums that bring together researchers, policy-makers, and sometimes beneficiaries of the results to inform and raise awareness for using research in decision-making. Further mechanisms of WHO regional offices to support this are regional scientific journals (eg, Eastern Mediterranean Health Journal) and programmes such as the Evidence-Informed Policy Network.

 Dissemination through publications, best practice guidance, and conferences are among the more traditional ways of sharing knowledge that regional organisations (eg, IGAD, CAMES) utilise. But multi-stakeholder platforms that bring decision-makers and researchers together was found to be the most common knowledge translation strategy used (by WAHO, ECSAHC [East, Central, and Southern Africa Health Community], IUCEA, AAS). For example, the ECSAHC sees its primary role as one of knowledge translation, by facilitating access of national policy-makers to research that responds to their policy challenges, such as through their Best Practices Forum. But discussions about evidence use are also part of their core business working with Ministers of Health in the ECSAHC annual meetings. Some organisations (eg, WAHO, IUCEA) have also used multi-stakeholder platforms to support innovation. For example, the IUCEA’s Academia Public-Private Partnership Forum is a platform that brings together universities, government, and private sector to create synergies and facilitate innovation and commercialisation.

####  Key Gaps – Production and Use 

 Many regional organisations have the authority and legitimacy to help facilitate platforms that convene and connect epistemic and policy communities. However, few of them have the mandate or capacity for coordinating or managing such multi-sectoral networks on an operational level unless supported through a long-term program. The regional organisations interviewed for our study also do not really have the mandate to produce knowledge, with a few specific exceptions. The majority generate data on specific themes through partnerships with universities or research institutions.

 The AU and WHO have been promoting evidence-informed decision-making for several years, and regional organisations generally reported that the forums they organise contribute to that agenda. The knowledge translation and dissemination work was reportedly carried out by regional bodies through specific platforms to increase research utilisation, but many informants expressed that this must be supplemented by advocacy for research use to government policy-makers. But informants recognised that there are still gaps in capacity for research use by policy-makers, noting room for improvement in advocacy. This ongoing advocacy is seen as fundamental from the perspective of regional organisations because multiple informants highlighted that the lack of understanding, prioritisation, and value of research by decision-makers is one of the major barriers to research use they encounter. The knowledge translation and policy platforms at the regional level should be supported by improving capacity within national institutions to use health research, such as through dedicated research synthesis units. However, informants underlined that the ability to use research also relies on the receptiveness of decision-makers and whether and how they consider research in their decision-making process.

###  Financing Health Sciences Research

 Financing represents the final core pillar explored, capturing funding for HSciR at the regional level, or contributions to funding schemes or programmes at the national level. We found fewer regional organisations involved in HSciR financing than in the other three pillars of health research systems. Even for organisations with a health mandate (eg, Africa CDC, WAHO, OCEAC), internal funding was marginal. Organisations generally sought funds from partners for research grants to in-country teams in member states or to conduct their own research; however, as reported above few have mandates for knowledge generation. Regional organisations in our study reported more indirect involvement in HSciR financing, through networking between their members and international donors and advocating for funding from national budgets of African governments.

 There are a few organisations contributing directly to funding HSciR in member states, but this is on a limited scale, except for AAS whose mandate is to fund and promote science. Technical organisations in health were found to be the main regional bodies providing HSciR financing, through small grants or facilitating access to research funds through collaborating partners. For example, several organisations (eg, African Development Bank [AfDB], Subregional Office for Eastern Africa, United Nations Economics Commission for Africa [SRO-EA/UNECA], IGAD) fund research projects on themes of interest, while WHO EMRO offers competitive research grants to countries in the region (which includes North African countries).

####  Key Gaps – Financing

 As an AU agency, AUDA-NEPAD was the only organisation interviewed that has the potential to reach and interact with wide range of government ministries other than health (ie, development, environment, finance) and heads of state. But there has been limited success in advocacy to convince governments to invest in HSciR, although some organisations reported that efforts to increase health sector budgets have seen some improvements. Nearly all informants cited dependence on foreign and external funds as an important barrier for ownership and local benefits of HSciR on the continent. International partnerships are also facilitators for HSciR capacity strengthening, but informants were concerned that reliance on these funds could have negative impact on long-term sustainability of independent researchers and research institutions in Africa.

 Regional organisations we interviewed expressed that regional economic communities should be more involved in mobilising alternative sources of funding to supplement public investments from governments and universities in HSciR. Several informants highlighted two important targets of advocacy for HSciR financing (other than governments) which they considered gaps and untapped resources, and which regional organisations are uniquely positioned to approach. The first is development finance institutions, such as bilateral and regional development banks, which have become increasingly interested in health. Yet, questions remain about how regional organisations can advocate convincingly to these finance institutions on behalf of the member states. The second new target for advocacy is the private sector and business. Informants from several regional organisations acknowledged that their engagement with the private sector as a source of investment in HSciR has been lacking despite the potential to do much more with this sector in Africa. One way to do this could be for regional organisations to work with member states to sensitise governments to the benefits of private sector investment in HSciR. Regional economic blocks could also help create a legal environment for private sector investment in national health research systems and private sector institutions as research producers. Several informants noted that large African corporations could contribute to financing HSciR in a sustainable way. For example, the UNITAID model (a multilateral initiative using airline tax to support research on HIV/AIDS) is one potential mechanism that might be adapted in the African context as innovative financing through the private sector. Organisations like AUDA-NEPAD, SRO-EA/UNECA, and AfDB have opportunities to advocate for economic development through innovation agendas, but they have not been actively fostering connections with private sector and industry in their activities with countries.

###  Cross-cutting Issues

Looking across regional organisations’ involvement in these pillars, we can identify several barriers and facilitators to their ability to help strengthen HSciR in countries such as the lack of prioritisation of HSciR at the national level, donor-driven HSciR priorities, histories of collaboration between groups of member states, and the internal institutional capacity of regional organisations to collaborate and work in HSciR. Some of these are also among important challenges for regional organisations previously identified in WAHO’s work to strengthen HSciR in West Africa.^35^ Three key cross-cutting issues emerge from our analysis as particularly important in influencing regional organisations’ involvement in strengthening HSciR in Africa.

####  Mandates Matter

 In analysing organisations’ activities related to strengthening the pillars of HSciR, institutional mandates and areas of authority of regional bodies were among the most common influential factors mentioned that affect their involvement in any given pillar. While many regional organisations share a general mandate to support integration, the policy areas that this extends to and the resources available to facilitate and maintain programmes to achieve that agenda, vary. Organisations with policy-area mandates related to line ministries responsible for governing HSciR at the national level (ie, health or education) seemed to have comparative advantages in pillars of governance, creating and sustaining resources, and using research. This technical expertise and mandate come through as important factors which are supported by their relationships and access to experts and decision-makers, including through policy and epistemic networks, in their member states in these policy fields.

 However, even when health is part of an organisation’s core mandate, there is no regional organisation in our study whose mandate is health research or HSciR (although it is integral to Africa CDC mandate to strengthen national public health capacity in Africa). The AU and UN organisations like Africa CDC, AUDA-NEPAD, and WHO regional offices are unique technical organisations, given their intergovernmental mandates for health or development across a large geographic scale (in the case of AU continent-wide). Each have different institutional designs with mechanisms for working with member states: Africa CDC through its 5 regional collaborating centres, AUDA-NEPAD through the regional economic communities, and WHO through direct work with countries via Ministries of Health and their country offices. This contrasts to the work carried out by regional bodies in sub-regional blocks with technical organisations in health and development who work directly with dedicated country representatives from ministries to their organisations and other institutions, like universities, in member states.

####  Power: Institutional Authority and State Sovereignty

 Related to mandates and institutional design, regional organisations were also found to exercise their authority to support HSciR in different ways. For example, many organisations exhibit epistemic power within their domains of expertise, as recognised and legitimate authorities in policy areas of health, development, education, or science. However, the expertise of regional organisations is moderated by constraints on their persuasive or coercive power to effect and enforce change based on their expert knowledge. Structurally, many of the regional organisations we interviewed are governed by member states, and as such, state-based regional cooperation relies on the decisions and voluntary actions of states, which can be a barrier since regional organisations do not have authority to enforce national implementation of decisions taken at the regional level. Many reported that the commitment of membership to regional work is necessary because state inaction or state action that does not align with regional priorities can hinder progress.

One of the main strengths of regional organisations is their convening power and access to decision-makers, which is an asset for advocacy. Many of them have direct access to Ministries of Health, Education, Science and Innovation, or Finance, as well as heads of state in some instances. This provides opportunities to influence political commitment, create dialogue, and mobilise African and international stakeholders. However, translating institutional legitimacy and prestige into action for strengthening HSciR at the national level has seen slow progress and with varied results. For instance, the development of the AUDA-NEPAD continental *Strategy for Health Research and Innovation in Africa*^34^ has demonstrated the epistemic and convening power of the organisation to engage with the regional economic communities, member states and other stakeholders to collectively set and agree on an agenda, but moving towards its implementation may require other forms of power (eg, persuasion, coercion) and cooperation that can leverage support, produce change, and foster collective action.

####  Collaboration: Trust, Shared Interests, National Priorities

 The final cross-cutting theme for regional organisations is collaboration with their member states and other stakeholders. Barriers to collaboration included the difficulty to work across partners and member states who have different, and sometimes competing priorities. The history of collaboration in a sub-region and strong networks between the countries were seen to be valuable foundations for proactive and sustainable approaches – especially when they can link up with the work of centres of excellence and research leaders in the sub-region – as seen in West Africa.

There was strong agreement in the data that collaboration with trusted partners had been vital because most of the work by regional bodies on HSciR is done through collaboration. Research on regional collaboration for HSciR in other regions across the global south has also shown the role of partnerships with national research institutions, international NGOs, development partners, and funding agencies to be critical to the success of regional organisations’ work to strengthen HSciR.^36-38^ Regional organisations noted that they can often be in the positions of brokering such collaborations between external partners and member states or African partners, and as such, they try to ensure those have mutual benefits for countries. However, dependence on these funds can risk concentrating HSciR in areas of interest to international partners, for which the outcomes do not necessarily address the priorities for the country or needs at a more local level. This is a concern for regional bodies which help to connect external funders with member states since these organisations can be used by interests from outside the region to influence African decision-makers.

## Discussion

 Our results on the role of regional organisations in relation to the four key pillars of health research systems have shown that organisations with technical mandates in or related to health reported being engaged in all pillars – with the most activity in governance and the use of health research. Regional organisations involvement in governance was mainly reported around setting regional agendas and policy harmonisation across member states. For those that reported contributing to the development of resources for HSciR, it was mainly through initiatives for strengthening human resources, with few involved in developing research institutions, networks, or infrastructure. Overall, regional organisations reported being more involved in dissemination than production of research. With respect to funding, regional organisations were more indirectly involved through facilitating contacts between funders and research teams or advocating member states to increase their budgets for HSciR. Regional organisations identified several gaps in activities where they believe their involvement should increase: better coordination within and across sub-regions, strengthening infrastructure for HSciR at the national or regional level, improved training and advocacy for research use, and engagement with the private industry sector and development institutions to increase financing of HSciR. Beyond the individual pillars, however, we also identified key cross-cutting themes in relation to mandates, authority, and collaboration that were particularly relevant to shaping the influence and activity of regional bodies on HSciR in Africa.

From our findings we identify three issues for regional cooperation in efforts to improve HSciR in Africa. First, more clarity is needed on the role of regional organisations (at the continental level and/or sub-regional level) in framing the agenda for strengthening HSciR in Africa. This raises questions about whether governance of HSciR at the regional level is top-down or bottom-up. Regional organisations work in different ways within the institutional landscape of health governance more globally, at the interface of the global arena and the national/local arena.^39^ In some instances, they serve as an intermediary within a top-down approach to governance and adapters of global standards, norms, and practices.^40,41^ In others, they act as a convenor of local or national expertise and interests to coordinate and advocate a bottom-up approach to health and rights.^3,42-44^ These are not mutually exclusive, and organisational behaviours and strategies may shift in response to internal or external factors. In both modalities, the proximity of regional organisations to a broad range of national stakeholders is an asset. For example, in this study we found intergovernmental organisations (eg, AUDA-NEPAD, WHO AFRO, Africa CDC) are developing strategies and frameworks for countries to adapt and align their national policies and programmes for health research systems. Yet, it is unclear whether these regional policies are intended for policy transfer and replication in countries, or whether they are rather intended as targets to set evaluation criteria against which progress in countries will be monitored by regional organisations. Organisations see their role as providing implementation support for these policies, but this is still lacking on a wide scale, with seemingly little being reported by regional organisations to support policy learning among countries and challenges to tracking the implementation and impact of regional policy decisions in individual countries.

Second, the ways that regional organisations build, support, or participate in networks for HSciR are unclear – whether that is in research networks or networking between regional organisations and other HSciR stakeholders in their region. Previous research on national health research systems has shown that regional research networks can be important mechanisms to foster research leadership and research culture, as well as generate advocacy for HSciR within countries.^18^ We spoke to only two organisations (WAHO, AAS) that reported actively and financially supporting the development of regional research networks in Africa. However, regional organisations recognised that a lot of research capacity development at the regional level is supported by regional networks, research platforms, and think tanks and often with collaboration of universities and other partners both within and outside Africa, like the African Population and Health Research Center. There is an opportunity for networking the networks that could fit within the broader integration mandates of regional organisations, to facilitate synergy for health research networks to interact and serve as key resources for regional organisations’ work with member states – especially given overlapping memberships when states belong to multiple regional organisations. Further, it may be important to consider the networks that regional organisations belong to as context for understanding their activities and roles in health and HSciR.

Third, there is incoherence in the lack of development of regulatory institutions for health research or science and innovation despite the larger efforts to harmonise regulation in sub-regions for select policy areas. Similarly, international funding is rarely available for regulatory capacity strengthening at the national level.^18^ Yet, developing statutory institutions for HSciR with regulatory and coordinating mandates (eg, national health research authorities) support an enabling environment, especially to integrate coordination between government authorities and research institutions.^18,45^ Relatedly, while many national decision-makers and researchers see the development of a national health research law as the gold standard for formalising the national health research systems^45^, none of the regional organisations in our study reported working with countries to support the development of a legal framework for HSciR.

There is emergent knowledge on the health policy agendas and programmes of regional bodies in Africa that when put in conversation with our findings, offers perspectives for further research. Notably, Yeates and Surender’s analysis of regional economic communities’ integration of health policy into their overall policy functions found that public health and healthcare is increasingly a strategic interest of these regional structures, but with differences in the way health policy is institutionalised and prioritised at the regional level.^46^ Although Yeates and Surender use a health systems framework for their analysis of health policy and programmes, there are analogous themes and cross-cutting issues to our findings from using a health research systems framework.

For instance, Yeates and Surender highlight that responsibilities for health within regional economic communities have not always been consolidated under Health Directorates (with the exception of SADC and IGAD), although some communities have their own regional health or health research organisations (eg, WAHO). One of their significant findings is the persistence of a siloed, vertical, and narrow approach to health within regional health policy and programmes without much emphasis on health system strengthening or universal healthcare.^46^ We suggest that this lack of integrated and cross-cutting approaches within the health domain at the regional level raises questions about institutional capacity of some regional organisations to address HSciR pillars across sectors. Our research has shown that health research systems at the national level are intersectoral by definition, involving health, higher education, and science, technology and innovation policy actors and stakeholders.^18^ So when health policy within regional organisations is structured by discrete issues or driven by funding of short-term projects, this may be a challenge for supporting health research systems which necessitates working across several-sectors within states. Regional organisations may only have ministerial counterparts and state representatives in a single sector (eg, finance, education, health). The means that it would be difficult to inform and advocate for regional level support and involvement in strengthening HSciR especially from member states with weak coordination of the national health research system. Ultimately, regional organisations are mainly governed by ministers and representatives of member states, while continental organisations vary more in terms of accountability mechanisms. Thus, the wishes and resources of member states are indispensable for the definition of regional priorities and their implementation. Based on results from both studies, one avenue of future research on why some regional organisations are not reporting any activities in health or health research to understand why member states are not using regional governance as a strategic venue to support their national systems.

Related to this, Yeates and Surender’s conclusion is critical of the health policy approach and discourse of regional economic communities as being framed more through an economic growth than human rights and social justice lens.^46^ Whereas, we found that the use of an economic growth frame for strengthening health research systems is desirable by many state actors as it provides an objective which links to national development plans that prioritise transitions to knowledge economies underpinning strategies being advanced by states for investment in research, development, and innovation.^18^ The results of the present study highlight the actual and desired role expressed by regional organisations for advocacy to improve sustainable financing for HSciR, and their framing of the need and multiple benefits for these investments will be a critical aspect of how they fulfil their role.

But there are clear signs of rapid advancements in regional policies and strategies related to health and health research facilitated as a consequence of health crises. Yeates and Surender note that several activities to strengthen regionalist approaches to health, like in regional disease surveillance, are relatively new since the West Africa Ebola outbreak of 2014/2015.^46^ Similarly, there have been multiple initiatives by the AU and Africa CDC since the beginning of the COVID-19 pandemic made possible through regional cooperation and coordination, to build regional efforts around preparedness and response to health crises, including strengthening HSciR.^47^

## Conclusion

 Within the literature on regional cooperation and health in Africa, this is one of the first attempts to identify and explore what regional organisations are doing to strengthen HSciR and what roles they play in supporting health research systems in Africa. Our findings show that many organisations are doing something related to supporting HSciR, but technical organisations with mandates or expertise in health or higher education policy fields seem to be most involved with activities across all key pillars of health research systems. Whilst regional organisations are not contributing much directly to financing HSciR, they are advocating for African governments to increase investment in HSciR. Future development of HSciR in the African continent should include strategic thinking about the roles, comparative advantages, and capability of regional organisations to facilitate HSciR capacity and growth. Regional bodies in Africa will no doubt play a key role in this, particularly in the wake of COVID-19. We hope this mapping and analysis can help contribute to future work in this important area.

## Acknowledgements

 All of the authors thank the participants in the study for their time and contributions.

 We also thank the LSE Global Health Initiative Peer Review Group for their critical review and discussion of the paper’s first revision. Their insights and feedback were valuable within our approach to the revision process.

 CMJ would like to acknowledge the SMASH Lab, a feminist research collective, online writing group. The consistent support and steadfast presence of this community of scholars during the analysis, writing, and revision of this paper was invaluable. I am grateful to share a reflective and nurturing space with them and for all that I learn from each one.

## Ethical issues

 The LSE Research Ethics Committee approved the study (REC ref # 757b), and informed consent was obtained before all interviews either verbally or in writing.

## Competing interests

 Authors declare that they have no competing interests.

## Authors’ contributions

 Conception and design of study: CMJ, CW, and JP. Acquisition of data: CMJ, JST, RM, and AH. Analysis and interpretation of data: CMJ, JST, RM, and JP. Design of interview guide and data analysis framework: CMJ, JST, RM, and JP. Drafting of manuscript: CMJ. Critical revision of manuscript: JST, RM, CW, and JP. Obtaining funding and Supervision: CW and JP.

## Funding

 This research was funded by Wellcome.

## Endnotes


[1] Since the time this research was conducted and data analysed in April 2021, with the paper submitted in May 2021, the AAS is no longer serving in this capacity as host and implementer of a financing platform due to an internal governance crisis.^48^ The Alliance for Accelerating Excellence in Science in Africa initiative has been re-established as the African Science Foundation in December 2021, temporarily hosted by Price Waterhouse Cooper in Kenya. Also as of December 2021, AUDA-NEPAD is host of the Coalition for African Research Initiative.


## Supplementary files


Supplementary file 1. Results From Stakeholder Mapping: Regional Organisations Supporting Health Sciences Research in Africa.

Supplementary file 2. List of Priority Organisations Identified for Interviews.

Supplementary file 3. Key Informant Interview Guide.

Supplemental file 4. Interview Coding Guide.
