# Supplementary material for: The Roles of Regional Organisations in Strengthening Health Research Systems in Africa: Activities, Gaps, and Future Perspectives
Source: Int J Health Policy Manag. 2022 Mar 8;11(11):2672–85. doi: 10.34172/ijhpm.2022.6426 (PMC9818106; doi:10.34172/ijhpm.2022.6426)
Supplement: Supplementary file 1 — Results From Stakeholder Mapping: Regional Organisations Supporting Health Sciences Research in Africa. [file ijhpm-11-2672-s001.pdf]

Article title: The Roles of Regional Organisations in Strengthening Health Research Systems in Africa: Activities, Gaps, and Future Perspectives

Journal name: International Journal of Health Policy and Management (IJHPM)

Authors' information: Catherine M. Jones[1]*, Joëlle Sobngwi-Tambekou[2], Rhona M. Mijumbi[3], Aaron Hedquist[4], Clare Wenham[1], Justin Parkhurst[1]

[1]Department of Health Policy, London School of Economics and Political Science, London, UK.

[2]Recherche-Santé & Développement (RSD Institute), Yaoundé, Cameroun.

[3]The Centre for Rapid Evidence Synthesis, College of Health Sciences, Makerere University, Kampala, Uganda.

[4]LSE Health, London School of Economics and Political Science, London, UK

(corresponding author: c.jones11@lse.ac.uk)

Supplementary file 1. Results From Stakeholder Mapping: Regional Organisations Supporting Health Sciences Research in Africa

Type of regional organisation	Principal Organisation	Sub-Organisation	Headquarters	Region
Technical (Health)	Africa CDC	Secretariat	Addis Ababa	Continent-Wide
Technical (Health)	Africa CDC	Central Africa Regional Collaborating Centre	Libreville	Central
Technical (Health)	Africa CDC	Eastern Africa Regional Collaborating Centre	Nairobi	Eastern
Technical (Health)	Africa CDC	Northern Africa Regional Collaborating Centre	Cairo	North
Technical (Health)	Africa CDC	Southern Africa Regional Collaborating Centre	Lusaka	South
Technical (Health)	Africa CDC	Western Africa Regional Collaborating Centre	Abuja	West
Technical (Science)	African Academy of Sciences	Secretariat	Nairobi	Continent-Wide
Technical (Science)	African Academy of Sciences	The Alliance for Accelerating Excellence in Science in Africa	Nairobi	Continent-Wide
Technical (Science)	African Academy of Sciences	Coalition for African Research and Innovation	Nairobi	Continent-Wide
Economic, Technical (Development)	African Development Bank	Secretariat	Abidjan	Continent-Wide
Economic	African Organisation for Standardisation		Nairobi	Continent-Wide
Technical (Science)	African Regional Intellectual Property Organization		Harare	South, East, Central, West

Technical (Science)	African Scientific, Research and Innovation Council		Abuja	Continent-Wide
Technical (Science)	African Union	African Observatory of Science Technology and Innovation	Oyala	Continent-Wide
Technical (Science)	African Union	Specialised Technical Committee on Health, Population, and Drug Control		Continent-Wide
Technical (Development)	African Union Development Agency	Secretariat	Midrand	Continent-Wide
Technical (Health)	African Union Development Agency	African Medicines Regulatory Harmonization Initiative		Continent-Wide
Technical (Health)	African Union Development Agency	Southern African Network for Biosciences	Pretoria	South
Technical (Health)	African Union Development Agency	Biosciences Eastern and Central Africa	Nairobi	East, South
Technical (Health)	African Union Development Agency	West African Network of Biosciences	Dakar	West
Technical (Health)	African Union Development Agency	North African Network for Biosciences	Cairo	North
Technical (Science)	African Union Development Agency	African Ministerial Council on Science and Technology	Pretoria	Continent-Wide
Technical (Science)	African Union Development Agency	Center of Excellence: Science, Technology and Innovation Hub	Pretoria	Continent-Wide
Political	African Union Commission	Secretariat	Addis Ababa	Continent-Wide
Economic, Political	Arab Maghreb Union		Rabat	North
Economic	Common Market for Eastern and Southern Africa		Lusaka	North, East, Central, South
Economic	Community of Sahel-Saharan States		Tripoli	North, East, West, Central
Technical (Development)	Conseil Africain et Malgache pour l'Enseignement Supérieur		Ouagadougou	West, Central, East
Economic	East African Community	Secretariat	Arusha	Central, East
Technical (Education)	East African Community	Inter-University Council for East Africa	Kampala	Central, East
Technical (Health)	East African Community	East African Health Research Commission	Bujumbura	Central, East
Technical (Health)	East African Community	East African Disease Surveillance Network	Arusha	East
Technical (Science)	East African Community	Science, Technology and Innovation Commission	Kigali	East
Technical (Health)	East, Central, and Southern Africa Health Community		Arusha	East, Central, South
Economic	Economic Community of Central African States		Libreville	Central, West
Economic	Economic Community of West African States		Abuja	West

Technical (Health)	Economic Community of West African States	West African Health Organisation	Bobo-Dioulasso	West
Political	Indian Ocean Commission		Port Louis	East
Economic	Intergovernmental Authority on Development		Djibouti	West
Economic	Organisation Africaine de la Propriété Intellectuelle		Yaoundé	Continent-Wide
Economic	Southern African Development Community		Gaborone	South, East, Central
Technical (Science)	Southern African Development Community	Science and Technology Desk	Gaborone	South, East, Central
Technical (Development)	United Nations	Economic Commission for Africa	Addis Ababa	Continent-Wide
Technical (Health)	World Health Organization	African Regional Office	Brazzaville	Continent-Wide
Technical (Health)	World Health Organization	Eastern Mediterranean Regional Office	Cairo	North
