# Supplementary material for: The Roles of Regional Organisations in Strengthening Health Research Systems in Africa: Activities, Gaps, and Future Perspectives
Source: Int J Health Policy Manag. 2022 Mar 8;11(11):2672–85. doi: 10.34172/ijhpm.2022.6426 (PMC9818106; doi:10.34172/ijhpm.2022.6426)
Supplement: Supplementary file 2 — List of Priority Organisations Identified for Interviews. [file ijhpm-11-2672-s002.pdf]

Article title: The Roles of Regional Organisations in Strengthening Health Research Systems in Africa: Activities, Gaps, and Future Perspectives

Journal name: International Journal of Health Policy and Management (IJHPM)

Authors' information: Catherine M. Jones[1]*, Joëlle Sobngwi-Tambekou[2], Rhona M. Mijumbi[3], Aaron Hedquist[4], Clare Wenham[1], Justin Parkhurst[1]

[1]Department of Health Policy, London School of Economics and Political Science, London, UK.

[2]Recherche-Santé & Développement (RSD Institute), Yaoundé, Cameroun.

[3]The Centre for Rapid Evidence Synthesis, College of Health Sciences, Makerere University, Kampala, Uganda.

[4]LSE Health, London School of Economics and Political Science, London, UK

(corresponding author: c.jones11@lse.ac.uk)

Supplementary file 2. List of Priority Organisations Identified for Interviews

Africa CDC

African Academy of Sciences

African Development Bank

African Regional Intellectual Property Organization

African Union

African Union Development Agency

Arab Maghreb Union

Common Market for Eastern and Southern Africa

Community of Sahel-Saharan States

Conseil Africain et Malgache pour l'Enseignement Supérieur

East African Health Research Commission (*East African Community*)

East, Central, and Southern Africa Health Community

Economic Community of Central African States

Intergovernmental Authority on Development

Inter-University Council for East Africa (*East African Community*)

Southern African Development Community

United Nations Economic Commission for Africa

West African Health Organisation (*Economic Community of West African States*)

WHO African Regional Office

WHO Eastern Mediterranean Regional Office
