# Supplementary material for: The Roles of Regional Organisations in Strengthening Health Research Systems in Africa: Activities, Gaps, and Future Perspectives
Source: Int J Health Policy Manag. 2022 Mar 8;11(11):2672–85. doi: 10.34172/ijhpm.2022.6426 (PMC9818106; doi:10.34172/ijhpm.2022.6426)
Supplement: Supplementary file 3 — Key Informant Interview Guide. [file ijhpm-11-2672-s003.pdf]

Article title: The Roles of Regional Organisations in Strengthening Health Research Systems in Africa: Activities, Gaps, and Future Perspectives

Journal name: International Journal of Health Policy and Management (IJHPM)

Authors' information: Catherine M. Jones[1]*, Joëlle Sobngwi-Tambekou[2], Rhona M. Mijumbi[3], Aaron Hedquist[4], Clare Wenham[1], Justin Parkhurst[1]

[1]Department of Health Policy, London School of Economics and Political Science, London, UK.

[2]Recherche-Santé & Développement (RSD Institute), Yaoundé, Cameroun.

[3]The Centre for Rapid Evidence Synthesis, College of Health Sciences, Makerere University, Kampala, Uganda.

[4]LSE Health, London School of Economics and Political Science, London, UK

(corresponding author: c.jones11@lse.ac.uk)

Supplementary file 3. Key Informant Interview Guide

Interview guide in English

Q1. What role do region organisations in Africa have in health sciences research?

Q2. Which of these roles is your organisation involved in?

NOTE: It is not necessary to ask all of the probes and follow up questions under Q2. These are here as reminders of the information that we want to collect, so that if it does not arise from the informant's reply to the open question, the interviewer can ensure to follow up on those areas that are priorities and probe for more information when needed. We want to focus on the main pillars, and a couple of processes that we know are key themes for regional cooperation from the former project.

Depending on the replies from informants to the open question here, interviewers should follow up with explicit questions about their organisation's own work directly on or in support of the four main **pillars** in other countries or with members. An informant's initial response may cover some of them. If they have mentioned any of these four in their reply, these can be followed up explicitly for deeper exploration. If not, the interviewer will ask about the other pillars not mentioned, because we want to be sure to have data about these.

The **process** themes may cut across the four pillars, as they may intersect with how the regional organisation is working in HSciR. It is not important to go through each process theme in every interview, unless it has been brought up by the informant or seems relevant to their replies about the role their organisation plays. Three processes are prioritised for data collection (advocacy, international partnerships and collaboration, and coordination) because these were highlighted in the former project as important advantages for regional cooperation and advantages. It is left to the discretion of the interviewer to ask about the other process themes specifically with regard to the key processes that regional organisations are involved in.

Probes and follow-ups for Q2:

P2.1. Where are your efforts to support health sciences research concentrated? In which country/ies is your organisation doing this?

<u>Main HSciR pillars</u> – to learn not only about what they are doing, but how they are doing it

P2.2 – Financing

How does your organisation finance health sciences research?

If further details needed on the how: What funding mechanisms does your organisation use for this? How are these funds used in countries?

P2.3 – Governance (regulation, policies, legislation, supervision/oversight)

How does your organisation participate in the governance of health sciences research at the national or regional level?

P2.4 – Creating and sustaining resources (human and institutional capacity, infrastructure)

How does your organisation contribute to strengthening capacity for health sciences research at the national or regional level? (e.g. support to individuals, universities and research institutions, Centres of Excellence, national/regional laboratories)

P2.5 – Producing and using health sciences research (knowledge translation, research use)

How does your organisation support the uptake of health sciences research results in the region?

<u>Key processes</u>* for HSciR – to learn about the processes used to carry out their work on HSciR

P2.6 – Advocacy

How does your organisation advocate for strengthening health sciences research in the region?

P2.7 – Collaboration

What kinds of partnerships and collaborations is your organisation involved in that support health sciences research in national settings or across the region?

P2.8 - Coordination

How does your organisation coordinate work in health sciences research with countries or with other organisations in the region?

*Other process themes for follow-up when relevant to the interview based on previous replies include: sustainability, alignment/prioritisation, sustainability, ownership/

Q3. What do you think has been the impact of your organisation’s work (through any of the roles you discussed above) to strengthen health sciences research in specific countries or the region more broadly?

If there is an example of impact or influence, then ask**:** How did your organisation achieve that influence?

Q4. Why has strengthening health sciences research (through any of the roles you discussed above) become a part of your organisation’s portfolio? (or why has it not?)

If further details needed on the how: What supported the inclusion of this in your organisation's activities? How did it support this?

Probes and follow-ups for Q4:

P4.1 - What policies or strategies does your organisation have to support health sciences research among your members or in the region?

P4.2 - What would make your organisation increase /or/ begin support of health sciences research in the region?

Q5. What have been the main facilitators to your organisation's work to strengthen health sciences research?

If further details needed on the how: How have these facilitated your organisation's work?

Q6. What have been the main barriers to your organisation's work to strengthen health sciences research?

If further details needed on the how: How have these hindered your organisation's work?

Q7. In your opinion, what should be the role of regional bodies to strengthen health sciences research in Africa? Why?

Q8. From your perspective, which are the most active and influential regional organisations that are strengthening health sciences research in specific African countries or any sub-regions?

Q9. Can you suggest any contacts in those organisations with whom we might speak?

Guide d'entretiens en français

Q1. Quel rôle jouent les organisations régionales en Afrique dans la recherche en sciences de la santé?

Q2. Dans lequel de ces rôles votre organisation est-elle impliquée ?

REMARQUE: Il n'est pas nécessaire de poser toutes les questions de suivi de la Q2. Il s'agit là de rappels de l'information que nous voulons recueillir, de sorte que si elle ne découle pas de la réponse de l'informateur à la question ouverte, l'intervieweur peut s'assurer de faire un suivi sur les domaines qui sont prioritaires et de sonder pour plus d'information en cas de besoin. Nous voulons concentrer sur les principaux piliers, et quelques processus du précédent projet que nous constatons sont des thèmes clés pour la coopération régionale.

Selon les réponses des informateurs à la question ouverte ici, les intervieweurs devraient donner suite avec des questions explicites sur le travail de leur organisation directement sur ou à l'appui des quatre principaux **piliers** dans d'autres pays ou avec les membres. La réponse initiale d'un informateur peut couvrir certains d'entre eux. S'ils ont mentionné l'un de ces quatre dans la réponse, ceux-ci peuvent être suivis pour une exploration plus approfondie. Sinon, l'intervieweur posera des questions sur les autres piliers qui ne sont pas mentionnés par l'informateur, parce que nous voulons être sûrs d'avoir des données à ce sujet.

Les thèmes du **processus** peuvent couper à travers les quatre piliers, et ils peuvent se croiser avec la façon dont l'organisation régionale travaille dans RSS. Il n'est pas important de passer en compte chaque thème de processus dans chaque entretien, à moins qu'il n'ait été soulevé par l'informateur ou semble pertinent à leurs réponses sur le rôle de leur organisation joue. Trois processus sont priorisés pour la collecte de données (plaidoyer, partenariats internationaux et collaboration, coordination) parce qu'ils ont été mis en évidence dans le premier projet comme des avantages importants pour la coopération régionale. Il est laissé à la discrétion de l'intervieweur de poser des questions sur les autres thèmes de processus (e.g. alignement, innovation, durabilité, propriété etc.) spécifiquement en ce qui concerne les processus clés dans les organisations régionales.

Sondes et suivis pour la Q2 :

P2. 1. Où se concentrent vos efforts pour soutenir la recherche en sciences de la santé? Dans quels pays votre organisation fait cela?

Principaux piliers de la RSS – pour en apprendre davantage non seulement sur ce qu'ils font, mais aussi sur la façon dont ils le font (comment ils le font)

P2.2 – Financement

Comment votre organisation finance la recherche en sciences de la santé ?

> Si d'autres détails sont nécessaires sur la façon dont ils le font: Quels mécanismes de financement votre organisation utilise-t-elle pour cela? Comment ces fonds sont-ils utilisés dans les pays?

P2.3 – Gouvernance (réglementation, politiques, législation, supervision/surveillance)

Comment votre organisation participe-t-elle à la gouvernance de la recherche en sciences de la santé au niveau national ou régional ?

P2.4 – Création et maintien de ressources (capacités humaines et institutionnelles, infrastructure)

Comment votre organisation contribue-t-elle au renforcement des capacités de recherche en sciences de la santé aux niveaux national ou régional ? (par exemple, soutien aux chercheurs, aux universités et aux établissements de recherche, aux centres d'excellence, aux laboratoires nationaux/régionaux)

P2.5 – Production et utilisation de la recherche en sciences de la santé (traduction des connaissances, utilisation de la recherche)

Comment votre organisation soutient-elle l'adoption ou l'utilisation des résultats de la recherche en sciences de la santé dans la région ?

Processus clés* pour la RSS – pour en apprendre davantage sur les processus utilisés pour effectuer le travail des organisations régionales sur la RSS

P2.6 – Plaidoyer

Comment votre organisation plaide-t-elle pour le renforcement de la recherche en sciences de la santé dans la région ?

P2.7 – Collaboration

Dans quels types de partenariats et de collaborations votre organisation participe-t-elle pour soutenir la recherche en sciences de la santé en milieu national ou dans la région?

P2.8 - Coordination

Comment votre organisation coordonne-t-elle le travail dans la recherche en sciences de la santé avec les pays ou avec d'autres organisations de la région ?

*D'autres thèmes de processus pour le suivi lorsqu'ils sont pertinents à l'entrevue sur la base de réponses précédentes comprennent: la durabilité, l'alignement,, la durabilité, la propriété

Q3. Selon vous, quel a été l'impact du travail de votre organisation (à travers l'un des rôles dont vous avez parlé ci-dessus) pour renforcer la recherche en sciences de la santé dans des pays spécifiques ou dans la région en général ?

> S'il y a un exemple d'impact ou d'influence: Comment votre organisation a-t-elle atteint cette influence?

Q4. Pourquoi le renforcement de la recherche en sciences de la santé (à travers l'un des rôles dont vous avez parlé ci-dessus) fait-il partie du portefeuille de votre organisation ? (ou pourquoi ne l'a-t-il pas fait?)

> Si vous en avez besoin détails sur le comment: Qu'est-ce qui a soutenu l'inclusion de cela dans les activités de votre organisation? Comment a-t-il soutenu cela?

<u>Sondes et suivis pour la Q4:</u>

P4.1 - Quelles politiques ou stratégies votre organisation a-t-elle pour soutenir la recherche en sciences de la santé parmi vos membres ou dans la région?

P4.2 - Qu'est-ce qui inciterait votre organisation à accroître /ou / à commencer à soutenir la recherche en sciences de la santé dans la région ?

Q5. Quels ont été les principaux facilitateurs du travail de votre organisation pour renforcer la recherche en sciences de la santé?

> Si d'autres détails sont nécessaires sur le comment: Comment cela a-t-il facilité le travail de votre organisation?

Q6. Quels ont été les principaux obstacles au travail de votre organisation pour renforcer la recherche en sciences de la santé?

> Si d'autres détails sont nécessaires sur le comment : Comment cela a-t-il entravé le travail de votre organisation?

Q7. À votre avis, quel devrait être le rôle des organismes régionaux pour renforcer la recherche en sciences de la santé en Afrique? Pourquoi ?

Q8. De votre point de vue, qui sont les organisations régionales les plus actives et influentes qui renforcent la recherche en sciences de la santé dans certains pays africains ou dans certains sous-régions?

Q9. Pouvez-vous suggérer des contacts dans les organisations avec lesquelles nous pourrions parler?
