# Supplementary material for: The Roles of Regional Organisations in Strengthening Health Research Systems in Africa: Activities, Gaps, and Future Perspectives
Source: Int J Health Policy Manag. 2022 Mar 8;11(11):2672–85. doi: 10.34172/ijhpm.2022.6426 (PMC9818106; doi:10.34172/ijhpm.2022.6426)
Supplement: Supplementary file 4 — Supplemental file 4. Interview Coding Guide. [file ijhpm-11-2672-s004.pdf]

Article title: The Roles of Regional Organisations in Strengthening Health Research Systems in Africa: Activities, Gaps, and Future Perspectives

Journal name: International Journal of Health Policy and Management (IJHPM)

Authors' information: Catherine M. Jones[1]*, Joëlle Sobngwi-Tambekou[2], Rhona M. Mijumbi[3], Aaron Hedquist[4], Clare Wenham[1], Justin Parkhurst[1]

[1]Department of Health Policy, London School of Economics and Political Science, London, UK.

[2]Recherche-Santé & Développement (RSD Institute), Yaoundé, Cameroun.

[3]The Centre for Rapid Evidence Synthesis, College of Health Sciences, Makerere University, Kampala, Uganda.

[4]LSE Health, London School of Economics and Political Science, London, UK

(corresponding author: c.jones11@lse.ac.uk)

Supplementary file 4. Interview Coding Guide

	Code	Description	Empirical*	Normative*	Impact*
**HSciR Pillar Codes**	1. Governance	When informant discusses the role of regional organisation in governance of HSciR.<br>- Legal framework for HSciR. This includes formal agreements and treaties.<br>- HSciR regulation. This includes the institutional structures, intellectual property, and national or regional regulation that guide HSciR.<br>- STI and development policies and priorities relevant for health research domain. This includes a broad range of policies that are tangentially related to HSciR such as STI, education, and potentially environment.<br>- HSciR policies, strategies, and priorities (i.e. institutional policies of regional organisation on HSciR, or those of organisation's members).<br>- Ethics and IRB initiatives, networks, and standards.	1.1	1.2	1.3

		- HSciR governance, norms and guidelines. This includes explicit initiatives or position papers on the governance of health research.			
	2. Infrastructure / Capacity	When informant discusses the role of regional organisation in infrastructure and human or institutional capacity strengthening of HSciR.<br>- Health research institutions, universities, HSciR collaborations, and national research centres, Centres of Excellence.<br>- National Laboratories.<br>- Research Management systems.<br>- Education, training, mentoring.	2.1	2.2	2.3
	3. Production / Use	When informant discusses role of regional organisation in producing and using HSciR.<br>- Involvement in knowledge production (projects, programmes).<br>- Access and availability of HSciR results.<br>- Knowledge translation platforms for knowledge use, application, adoption, evaluation.<br>- Visibility and dissemination (including scientific publications and conferences, multi-stakeholder platforms, and the public).	3.1	3.2	3.3
	4. Financing	When informant discusses role of regional organisation in financing HSciR.<br>- Regional health research funds.<br>- National funding schemes.<br>- Resource mobilisation and financing instruments.	4.1	4.2	4.3
**Processes Codes**	5. Advocacy	When informant discusses what advocacy the regional organisation does or should do for HSciR.<br>Ability and arguments to communicate with and convince policy-makers and politicians for research support and research use (about relevance/significance of HSciR and for any of the pillars of HSR).	5.1	5.2	

	6. Collaboration	When informant discusses what collaboration the regional organisation engages in or should engage in for HSciR.<br>- Partnerships and collaborations with other stakeholders (national governments, international organisations, regional organisations, NGOs, funders, universities, private sector industry, communities).<br>- Networks/networking - North-South, South-South, Anglophone-Francophone, African region (continent), sub-regional. Conferences, seminars, or other partner meetings, and networking platforms in the region.	6.1	6.2	
	7. Coordination	When informant discusses what coordination the regional organisation does or should do for HSciR.<br>- Coordination institutions/structures, mechanisms, arrangements. Information management.	7.1	7.2	
	8. Alignment	When informant discusses what alignment or harmonisation the regional organisation does or should do for HSciR.<br>- Alignment of HSciR with the national or regional contexts, e.g. linking the strategic visions to empirical realities on the ground, linking HSciR to population needs / health priorities, linking HSciR to goals for improving health and/or health systems, linking HSciR to development (economic or social) and/or innovation.<br>- Alignment of HSciR with other policies, programmes, strategies (health, development, etc.) at local, national, regional and/or international levels.	8.1	8.2	
	9. Innovation	When informant discusses what regional organisations does or should do for innovation of HSciR.<br>- The application of research ideas for developing new products and technologies.<br>- Creation of new business, products, services from HSciR (e.g. patent development).	9.1	9.2	
**Key Issues**	10. Ownership	When informant discusses what the regional organisation does or should do for improving and increasing national or regional ownership of HSciR.	10.1	10.2	

	11. Sustainability	When informant discusses what the regional organisation does for improving and increasing sustainability of HSR at the regional or national level.	11.1	11.2	
**Key Barriers and Supports**	12. Barriers	Main barriers to the regional organisation's work to strengthen HSciR. Factors which hinder the regional organisation's work.			
	13. Facilitators	The main facilitators to the regional organisation's work to strengthen HSciR. The factors which have supported the regional organisation's work.			
	14. Motivation	The reasons why the regional organisation has included work to strengthen HSciR as part of its portfolio (or why has it not?). The factors that support, or the rationale for, the inclusion of this in the regional organisation's activities.			
**Other**	15. Other	When using the "other" code, insert a note or attach a linked memo to specify what the code theme is and why it is important to highlight as an emergent theme.			
	16. Great quote	Strong quote that communicates clearly an idea or theme very well.			
***When applicable, interviews were coded along three domains:**<br>1) Empirical - informants perceive their organisations' activities and involvement in HSciR;<br>2) Normative - how informants believe regional organisation should be engaging with HSciR;<br>3) Impact - where informants perceive their regional organisation has had impact in HSciR.					
